# Identification of Reproduction-Related Gene Polymorphisms Using Whole Transcriptome Sequencing in the Large White Pig Population

**DOI:** 10.1534/g3.115.018382

**Published:** 2015-04-27

**Authors:** Daniel Fischer, Asta Laiho, Attila Gyenesei, Anu Sironen

**Affiliations:** * Natural Resources Institute Finland (Luke), Green Technology, Animal and Plant Genomics and Breeding, FI-31600 Jokioinen, Finland; † The Finnish Microarray and Sequencing Centre, Turku Centre for Biotechnology, University of Turku and Åbo Akademi University, Tykistökatu 6, FI-20520 Turku, Finland; ‡ Campus Science Support Facilities, Vienna Biocenter, A-1030 Vienna, Austria

**Keywords:** oviduct, testis, gene expression, polymorphism, SNP, pig, transcriptome, RNAseq, reproduction

## Abstract

Recent developments in high-throughput sequencing techniques have enabled large-scale analysis of genetic variations and gene expression in different tissues and species, but gene expression patterns and genetic variations in livestock are not well-characterized. In this study, we have used high-throughput transcriptomic sequencing of the Finnish Large White to identify gene expression patterns and coding polymorphisms within the breed in the testis and oviduct. The main objective of this study was to identify polymorphisms within genes that are highly and specifically expressed in male and/or female reproductive organs. The differential expression (DE) analysis underlined 1234 genes highly expressed in the testis and 1501 in the oviduct. Furthermore, we used a novel in-house R-package hoardeR for the identification of novel genes and their orthologs, which underlined 55 additional DE genes based on orthologs in the human, cow, and sheep. Identification of polymorphisms in the dataset resulted in a total of 29,973 variants, of which 10,704 were known coding variants. Fifty-seven nonsynonymous SNPs were present among genes with high expression in the testis and 67 were present in the oviduct, underlining possible influential genes for reproduction traits. Seven genes (*PGR*, *FRAS1*, *TCF4*, *ADAT1*, *SPAG6*, *PIWIL2*, and *DNAH8*) with polymorphisms were highlighted as reproduction-related based on their biological function. The expression and SNPs of these genes were confirmed using RT-PCR and Sanger sequencing. The identified nonsynonymous mutations within genes highly expressed in the testis or oviduct provide a list of candidate genes for reproduction traits within the pig population and enable identification of biomarkers for sow and boar fertility.

During recent years, genetic studies have revealed an increasing number of associated markers and causative genes related to production and reproduction traits in livestock [Online Mendelian Inheritance in Animals (OMIA) (Lenffer *et al.* 2006) and The Animal Quantitative Trait Loci (QTL) Database (Animal QTLdb) (Hu *et al.* 2013)]. Genetic markers have proved especially important for traits whose measurement is difficult, expensive, only possible late in life in the study subjects, sex-limited, or not possible on selection candidates (Davis and Denise 1998). Advances in molecular genetics have led to the identification of several gene polymorphisms that have an economic impact on animal production (Clop *et al.* 2006; Grisart *et al.* 2002; Milan *et al.* 2000; Murphy *et al.* 2006; Sironen *et al.* 2006, 2011; van Laere *et al.* 2003). Recent developments in high-throughput sequencing techniques such whole transcriptome sequencing (RNAseq) have enabled large-scale analysis of genetic variations and gene expression in different tissues and species. Several studies of the transcriptome in humans and model species have increased our knowledge of tissue-specific gene expression. In addition, previous studies have provided some foundation for the transcriptional landscape of the pig based on microarray technology (Freeman *et al.* 2012). Transcriptomic analysis of pig reproductive organs (the placenta and testis) (Du *et al.* 2014; Esteve-Codina *et al.* 2011) using RNAseq have provided further insights in the gene expression of these tissues. However, the gene expression patterns and variations in livestock species remain poorly understood. Although the reference genome is available for the pig, enabling the identification of candidate genes for various traits, detailed annotation and identification of tissue specific expression patterns still require additional studies. Transcriptome sequencing also enables comprehensive analysis of gene isoforms and novel genes.

In this study we have used high-throughput transcriptomic sequencing of the Finnish Large White breed to identify gene polymorphisms and expression patterns related to reproduction. The dataset contains sequences from samples collected from immotile short-tail sperm defect (ISTS)-affected individuals and control animals. ISTS-affected boars are infertile due to immotile and short sperm tails (Andersson *et al.* 2000; Sukura *et al.* 2002). The ISTS phenotype is caused by an altered splicing pattern of exon 30 of the *SPEF2* gene, which results in premature translation stop codons (Sironen *et al.* 2006). The cause for the altered splicing pattern was shown to be a full-length L1 insertion within intron 30 (Sironen *et al.* 2006, 2007). We have investigated and identified an association between the L1 insertion and litter size in the Finnish Large White pig population (Sironen *et al.* 2012), which may be caused by a significant decrease in *PRLR* expression in the ISTS-affected and carrier sows (Sironen *et al.* 2014). Because the mechanisms underlying the causative mutation are known, we assume that the ISTS mutation does not induce changes in the expression of other genes between the ISTS-affected and control animals. The present investigation tested the hypothesis that RNAseq data from two testis and two oviduct samples can be used, first for the identification of highly expressed genes in these tissues, second for discerning genes specifically affecting male or female reproduction, and third for characterizing gene polymorphisms within identified genes. These genetic polymorphisms serve as candidate variants in association analysis and consequently potential biomarkers for pig fertility. Furthermore, we have used the data for the discovery of 55 previously unannotated genes using a novel in-house analysis pipeline.

## Materials and Methods

### Animal material

Tissue samples of the testis and oviduct from Finnish Large White pigs were collected at slaughter. The dataset contains samples of ISTS-affected and control animals. Previously, we have studied the effect of ISTS on gene expression within the ISTS-associated region (Sironen *et al.* 2006, 2007, 2014), and we have shown that only the expression of *SPEF2* and *PRLR* are affected. At slaughter, sows were approximately 4.5 months old and had not been bred. Boars were mature and had been used for breeding. The oviduct and testis tissue samples were collected in RNAlater buffer (Qiagen) and stored at −80°. For RNAseq analysis, four samples were used: the oviduct from an ISTS homozygous sow and a control sow, and the testis from an ISTS homozygous boar and a control boar. For RT-PCR, additional samples of two ISTS and control sows and boars were collected.

### RNA extraction and library preparation

Total RNA was extracted using RNeasy Midi kit (Qiagen) following the manufacturer’s instructions. The quality and concentrations of the RNA were checked using the Agilent 2100 Bioanalyzer (Agilent) and Nanodrop ND-2000 spectrophotometer (Thermo Scientific). Ribosomal RNA was removed with Ribominus^TM^ Eukaryote Kit for RNAseq (Invitrogen), and ribosomal RNA-depleted total RNA was fragmented using RNaseIII, to convert the whole transcriptome sample to RNA of a size appropriate for SOLiD System sequencing. After clean-up using the Purelink^TM^ RNA Micro kit, fragmented RNA samples with sufficient yield and an appropriate size distribution were ready for preparation of amplified cDNA libraries. Quality of the fragmentation was checked with the Bioanalyzer. The fragmented RNA sample was hybridized and ligated with the Adaptor Mix. RNA population with ligated adaptors was reverse-transcribed to generate single-stranded cDNA copies of the fragmented RNA molecules. After a clean-up step using the MinElute PCR Purification Kit, the sample was subjected to denaturing gel electrophoresis, and gel slices containing cDNA of the desired size range were excised. The size-selected cDNA was amplified using 15 cycles of PCR that takes place in the gel slices. This step appends required terminal sequences to each molecule and generates a sufficient template for SOLiD sequencing. After PCR, the amplified cDNA was cleaned using the PureLink^TM^ PCR purification kit. Libraries were quantitated with two different methods: Qubit fluorometer (Invitrogen) and quantitative PCR to ensure accuracy. The SOLiD Library TaqMan Quantitation Kit was used for determining the molar concentration of amplified template in a SOLiD library. In qPCR, the standard and unknown library template are amplified using two sequence-specific primers with a TaqMan fluorogenic probe labeled with FAM dye and a dye quencher. Uniformity of fragment size of libraries was confirmed with the Bioanalyzer. Templated bead preparation was performed by emulsion PCR (ePCR). SOLiD EZ Bead instrumentation was used for templated bead preparation.

### Transcriptomic data analysis

The colorspace reads obtained from the SOLiD sequencer were aligned against the pig reference genome (Sus scrofa build 10.2) using the standard whole transcriptome pipeline and the colorspace alignment tool provided by Applied Biosystems and supplied with the instrument (LifeScope v2.1). Reads associated with ribosomal RNA, transfer RNA, repeats, and other uninformative reads were filtered out during the process, as were reads with more than 10 potential alignments. For mapped reads, two mismatches per split were allowed, with two valid adjacent mismatches, which are likely to be SNPs, and were counted only as a single mismatch. After alignment to the reference genome, low-mapping-quality reads were discarded [mapQV (<10)] and unique reads were associated with known genes based on UCSC annotations, and the number of reads aligned within each gene was counted. FPKM values were calculated following normalization of the data to remove variation between samples caused by nonbiological reasons, including library size and gene length using the Cufflinks software (v2.0.2) (Trapnell *et al.* 2012). Thus, the values generated are independent of the total number of reads in each sample and make the data comparable across the sample set. For differential expression analysis with the Cufflinks software, an FDR of no more than 0.01 and a minimum log fold-change of two was set as a threshold for a gene to be considered differentially expressed (DE) between groups.

Gene classification was performed with the Panther (Protein ANalysis THrough Evolutionary Relationships) classification system (Mi *et al.* 2005; Thomas *et al.* 2003). GO enrichment analyses of the Cufflinks results were performed using AgriGO (Du *et al.* 2010) and GOrilla (Eden *et al.* 2009). AgriGO accepted only protein IDs, which limited the power of the analysis due to a low number of identified genes with the correct ID. Thus, the knowledge of human gene function was exploited for the identification of enriched biological processes and classification analysis. For human genes, the list of all Ensembl annotated genes was used as the reference list. Associated gene names were obtained for pig genes by Biomart.

### Gene variant detection

For identification of polymorphisms in the Finnish Large White transcriptome, we used the Genome Analysis Toolkit (GATK) (Depristo *et al.* 2011; McKenna *et al.* 2010; van der Auwera *et al.* 2013) program and SNP and Variation Suite v7 (Golden Helix, Inc., Bozeman, MT; www.goldenhelix.com) for filtering and annotation of the identified variants. The data were filtered based on the read depth (>5), genotype quality (>20), and quality score (>80). The possible effect of amino acid substitutions on protein function was analyzed using SIFT (http://www.ensembl.org/Tools/VEP), and the effect on the secondary structure of the protein was analyzed using the CFSSP (http://www.biogem.org/tool/chou-fasman/) prediction tool.

### Gene ortholog detection

Novel transcripts detected by the Cufflinks suite were used to identify genetically active, but so far unannotated, regions in the Sus Scrofa genome. Detected sequences that were longer than 130 bp and that had a FPKM value of at least 5 for at least one of the samples were blasted against full genomes in the NCBI database “chromosomes” using the in-house R-package hoardeR (http://cran.r-project.org/web/packages/hoardeR/index.html). Hits that had an identity ratio larger than 0.9 were processed further. Due to the large amount of hits, subsequent analysis focused on hits in the top three species (bos taurus, homo sapiens, and ovis aries). The genome assemblies of the top species used were as follows: Homo sapiens, CRCh38; Bos Taurus, UMD3.1; and Ovis Aries, OAR3.1. Hits were then cross-checked against the gene annotations of the species so that the hits could be grouped into intergenic, intronic, and exonic hits. The R-package edgeR (Robinson, McCarthy and Smyth 2010) function exactTest was then applied for differential expression testing between the oviduct and testis samples and intronic/exonic hits. Values of FDR less than 0.01 were considered to be differentially expressed.

Genomic regions with multiple hits within single genes were further investigated using a pairwise comparison approach based on sliding windows. Setting the window size to the smallest hit length, the genomic sequences of Sus Scrofa and the hit organism were divided into chunks of this window size, followed by a pairwise sequence similarity comparison between all chunks. The pairs with the largest similarities were reported and, for visualization of the region, indicated as a colored bar using a color spectrum between red and green for the degree of similarity. Pairs with low similarity (<0.3) were omitted from the corresponding visualizations.

### RT-PCR and sequencing

For analysis of gene expression with RT-PCR, RNA of the testis and oviduct samples from two controls and ISTS homozygous animals were extracted (RNeasy Midi kit; Qiagen). Total RNA was reverse-transcribed with random primers and an RT-PCR kit (ImProm-II Reverse Transcription System; Promega) according to the manufacturer’s instructions. Synthesized cDNA was amplified using gene-specific primers (Supporting Information, Table S1). The housekeeping gene ribosomal S18 *(RIBS18)* was used as a reference gene to calculate the relative expression. cDNA samples were diluted to 20 ng/μl prior to use. The qPCR was performed with a ViiA 7 Real-Time PCR System in 96-well microtiter plates using Absolute qPCR SYBR Green ROX Mix (VWR). Amplification by qPCR contained 12.5 μl of Absolute qPCR SYBR Green Mix, 100 ng of cDNA, and 70 nM of each primer in a final volume of 25 μl. Amplifications were initiated with 15 min of enzyme activation at 95°, followed by 40 cycles of denaturation at 95° for 15 sec, primer annealing at 60° for 30 sec, and extension at 72° for 30 sec. All samples were amplified in triplicate, and the mean value was used in further calculations. Raw data were analyzed with the sequence detection software (Applied Biosystems) and relative quantitation was performed with GenEx software (MultiD). Ratios between the target and reference gene were calculated using the mean of these measurements. A standard curve for each primer pair was produced by serially diluting a control cDNA and used to correct for differences in amplification. A melting curve analysis was performed allowing single product-specific melting temperatures to be determined. No primer–dimer formations were generated during the application of 40 real-time PCR amplification cycles.

For sequencing, the RT-PCR amplicons were purified using ExoSAP-IT (Amersham Biosciences). PCR fragments were sequenced in both directions with the same primers used for amplification. Sequencing was performed on a MegaBace 500 capillary DNA sequencer (Amersham Biosciences) using DYEnamic ET Terminator Kits with Thermo Sequenase II DNA Polymerase (Amersham Biosciences).

## Results and Discussion

### Data evaluation

The Life Technologies’ SOLiD 4 sequencing platform was used for transcriptomic sequencing of the porcine testis and oviduct samples. Approximately 70 to 80 million reads were obtained for each sample (Table 1). Between 30% and 65% of the reads could be mapped against the pig genome build 10.2. Pearson’s correlation between the testis samples was 0.94, and was 0.76 between the more heterogeneous oviduct samples. Hierarchical clustering using Euclidean metrics and average linkage method grouped the parallel tissue samples clearly together as expected. After filtering low-mapping-quality reads [mapQV (<10)], most of the reads were mapped on exons (27–41%), and many were mapped on intergenic regions (25–33%) (Table 1), indicating potentially unannotated expressed genes in the pig genome.

**Table 1:** Mapping statistics

	Total Reads	Mapped Reads	Mapping %	Counted on Exons	Counted on Introns	Counted on Intergenic Regions	Exon %	Intron %	Intergenic %
ISTS testis	81,425,334	53,794,961	66	20,498,025	2,187,936	16,451,710	38.1	4.1	30.6
WT testis	68,655,352	20,507,264	30	5,995,191	1,177,538	5,882,955	29.3	5.7	28.7
ISTS oviduct	76,914,157	45,794,708	60	11,817,207	1,999,766	18,434,196	25.8	4.4	40.3
WT oviduct	72,020,521	44,993,220	63	10,457,619	2,467,954	17,987,423	23.1	5.5	40.0

Uncounted reads failed the mapping quality filter.

### Identification of candidate genes for reproduction in the pig testis and oviduct

For calculation of the normalized gene expression values, we used the Cufflinks software. In total, 16,595 (FPKM >0.2) genes were expressed in the testis and 15,846 genes were expressed in the oviduct. Genes with the highest expression (top 80; Table S2) in the testis were associated with spermatogenesis as expected (Figure S1A). In the oviduct, the top 80 genes highlighted reproduction, regulation of system process, embryonic development, regulation of actin polymerization, and translational elongation (Figure S1C). A total of 27 genes had high expression in both the testis and oviduct (Figure S1B). These genes were not enriched significantly toward any GO terms, but many of them (n = 15) were associated with metabolic processes (Figure S1D).

### Differentially expressed genes in the testis and oviduct

**Figure 1:**
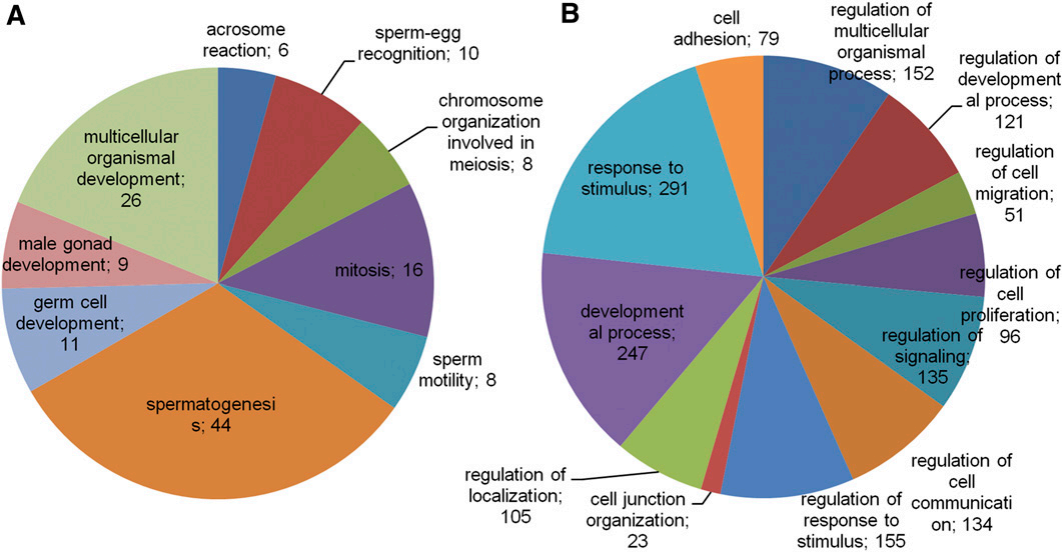
Enriched GO terms among upregulated genes (*P* < 10^−5^). (A) Enriched biological processes among upregulated genes in the testis. (B) Enriched biological processes among upregulated genes in the oviduct.

The analysis of gene expression differences between the testis and oviduct highlighted 1234 upregulated genes in the testis and 1501 in the oviduct. Significantly enriched GO terms (*P* < 10^−5^) in the testis included acrosome reaction, sperm–egg recognition, sperm motility, spermatid development, cell-cycle process, and spermatogenesis (Figure 1A). In the oviduct, more diverse processes were significant, including signaling, regulation, and developmental processes (Figure 1B). The top 30 DE genes based on fold change (FC) are listed in Table S3. Genes with a high FC between the testis and oviduct resulted in enrichment in sperm motility (*SMCP*, *TNP1*), spermatogenesis (*INSL3*, *PRM1*, *ADAM29*, *SPEM*), and histone exchange and nucleosome disassembly (*HIST1H2BA*, *TNP1*) in the testis and in muscle contraction (*DES*, *TPM2*, *TNNC1*, *ACTG2*), extracellular negative regulation of signal transduction (AGTR2, *LTBP1*), and mesenchymal-epithelial cell signaling (*TNC*, *HOXA5*) in the oviduct. Several pseudogenes (*LOC100510878*, *LOC100516119*, *LOC100519930*, and LOC100626054) and ncRNA (*LOC100523888*, *Spty2d1-AS1*) were also specifically expressed among the top 30 expressed genes in the testis. In total, 47 genes with a known role in reproduction were upregulated in the oviduct and 52 genes were upregulated in the testis (Table S4). These reproduction-related genes appear to be sex-specific, underlining possible candidate genes for reproductive performance in the pig. In total, 440 genes with a biological role in reproduction were identified in the dataset based on human gene names. Gene names were identified and recognized by Panther tool for 10,320 expressed genes.

### Novel transcripts and gene orthologs

**Figure 2:**
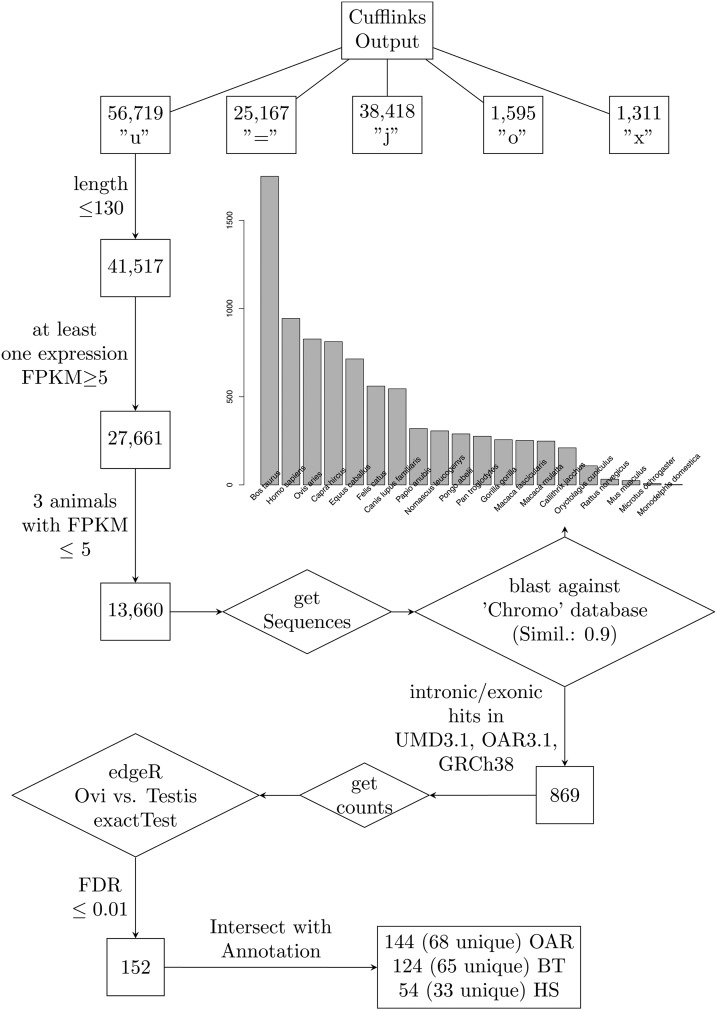
Flowchart of the analysis pipeline for novel transcripts. Several filtering steps were included for the identification of previously unannotated exons using a blast search against the available genomes in the NCBI chromosome database. The number of hits for each species is shown in the bar chart. Species with the highest number of hits (cow, human and sheep) were selected for identification of novel DE genes between the testis and oviduct in the pig.

Cufflinks predicted a large number of novel single-exon transcripts (56,719). During the mapping of sequence reads using Life Technologies LifeScope software, spliced reads were only included for the annotated genes, which prevented the construction of novel multi-exon transcripts. After filtering the novel transcripts according to a minimum length (>130 bp) and FPKM value (>5), a total of 13,660 novel candidate transcripts were identified (Figure 2). Furthermore, Cufflinks predicted 38,418 new isoforms, 1595 transcripts with generic overlap with reference genes, and 1311 transcripts with exonic overlap with reference on the opposite strand (Figure 2). For 448 of these opposite strand transcripts in the oviduct and for 787 in the testis, the FPKM was >1. Functional analysis of these genes did not indicate any enriched GO terms, but they do represent possible regulatory sequences for expressed genes in the testis and oviduct.

**Figure 3:**
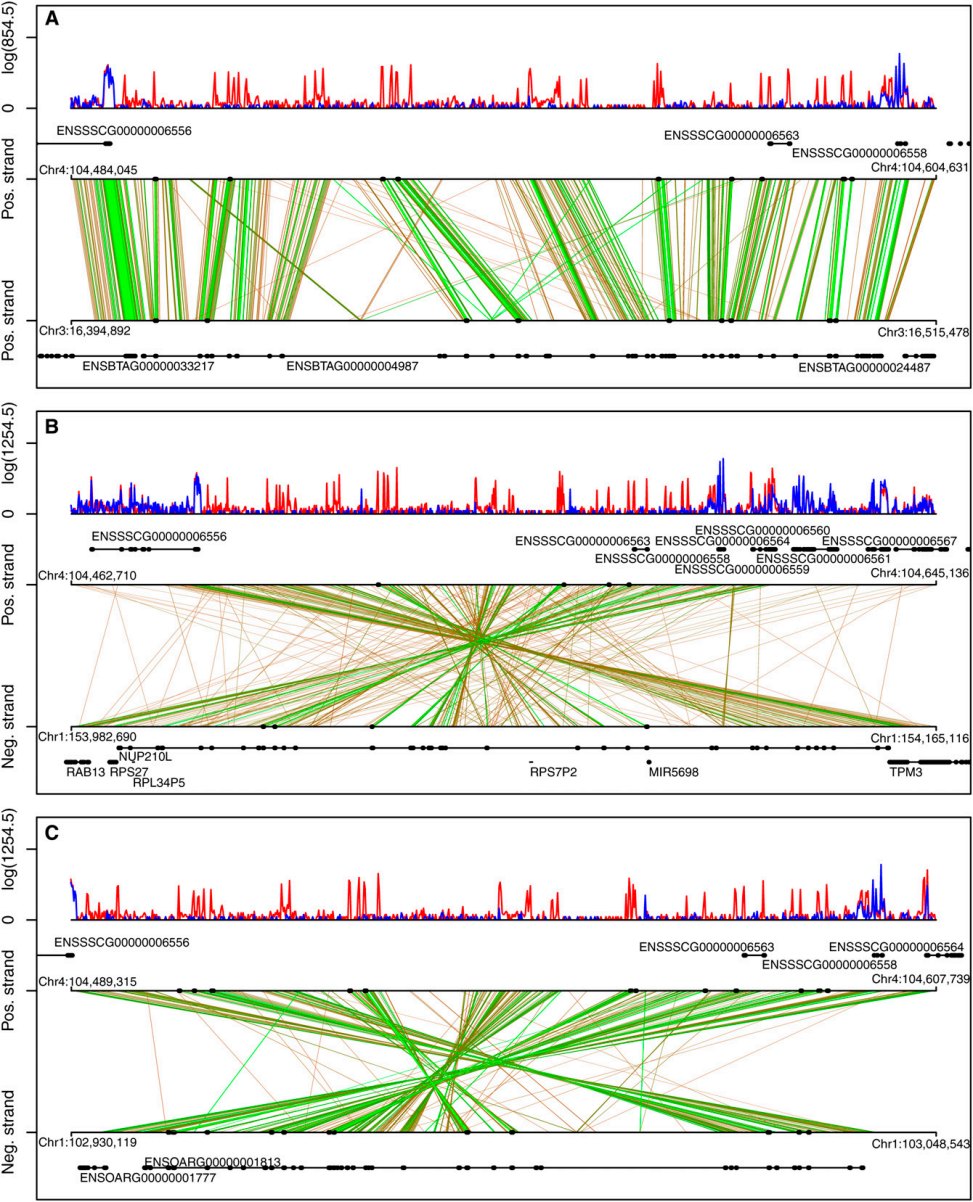
Conserved genomic sequences of *NUP210L* between pig and cow, human, and sheep. (A) Comparison of the *NUP210L* genomic region between the pig and cow. (B) Comparison of the *NUP210L* genomic region between the pig and human. (C) Comparison of the *NUP210L* genomic region between the pig and sheep. Log-expression levels (FPKM) are shown as red peaks in the testis and as blue in the oviduct, and the similarity of the hit is indicated with the deepening shades of green. Annotation of the genes in the pig is presented above the alignment, and those for the human, cow, or sheep are shown below the alignment.

**Figure 4:**
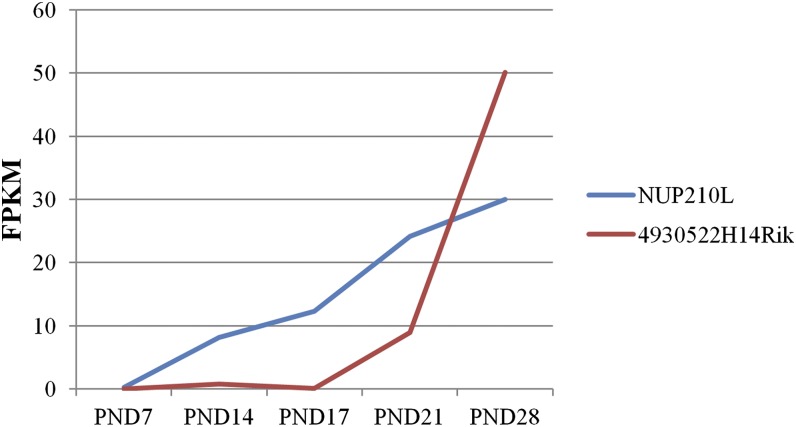
Expression of *NUP210L* and 4930522H14Rik (C3H1orf185/C1orf185) during the first wave of spermatogenesis in the mouse.

For the identification of gene orthologs in the dataset, we ran a blast search against the genomic sequences of all species available in the NCBI “chromosome” database. Most hits (similarity >90%) were identified in the Bos Taurus assembly UMD3.1 (n = 888). The alignment of genomic sequences between the pig and three other mammalian species (human, cow, and sheep) with the highest amount of sequence hits showed high coherency between corresponding chromosomes (Figure S2, A–C). The amount of novel and common hits between the top three species is shown in Figure S2D. Novel gene orthologs with intronic or exonic hits in these genomes (n = 869) were selected for differential expression analysis between the testis and oviduct, which resulted in 152 DE hits (FDR ≤0.01). These hits were selected based on the criterion that the hit sequence was completely included in the exon or intron of an annotated gene. The number of exonic hits increased considerably when partial exon hits were included (Figure S2E). In total, DE hits were identified in 55 unique genes. Most hits were assigned to nucleoporin 210kDa-like (*NUP210L*) gene (Table S5 and Figure 3). *NUP210L* appears to be testis-specific and probably has a role in spermatid development. Annotation of the *NUP210L* gene is incomplete in the pig genome (10.2.73), where only 2 out of 40 exons in the human (Ensembl database) are annotated. Based on our expression data, most annotated exons in the human, cow, and sheep are also expressed in the pig testis (Figure 3, A–C). Thirty-five genes in the testis and 20 in the oviduct showed high expression (Table S5). These included some recent annotations, which underline possible novel reproduction-related genes. To elucidate the possible role of these genes, we investigated the expression pattern during the first wave of mouse spermatogenesis based on our previous data (Laiho *et al.* 2013) in which a mouse ortholog was identified. Two genes with mouse ortholog 4930538K18Rik (ENSBTAG00000017387/ENSOARG00000020461) and 4930522H14Rik (C3H1orf185/C1orf185) showed increased expression during spermatogenesis, with the highest mRNA level at postnatal day (PND) 28 (Figure 4). Each time point corresponds to the appearance of specific cell types in the collected tissue sample: spermatogonia at PND 7; early pachytene spermatocytes at PND 14; late pachytene spermatocytes at PND 17; round spermatids at PND 21; and elongating spermatids at PND 28 (Laiho *et al.* 2013). 4930522H14Rik appeared to be testis-specific in the mouse, and 4930538K18Rik showed high expression in the oviduct and testis (http://www.ncbi.nlm.nih.gov/UniGene). This expression pattern indicates a role for these genes in late steps of spermatid elongation. The expression of NUP210L also increased during the first wave of spermatogenesis (Figure 4).

### Genetic variations in the Finnish Large White

After the filtering steps, a total of 29,973 variants were identified in the dataset, of which 10,704 were known coding variants (Table 2). These included 1672 nonsynonymous variants, 8 additional stop codons, a loss of 7 stop codons, 3436 unknown variants, and 1194 splicing variants. However, some of the splicing variants arise from mapping errors and therefore require confirmation prior to use in further experiments. Two of the stop loss variations have been previously reported, but 13 appear to be novel (Table 3). After detailed analysis, the variation within *MCL1* appears to be an annotation and splicing error. Based on the comparison of the predicted protein sequence translated from our expressed sequences (Figure S3A), the pig *MCL1* protein sequence (ENSSSCP00000007094), and human *MCL1* protein (ENSP00000358022), the annotation of the pig *MCL1* is incomplete and the protein prediction is incorrect (Figure S3B). Furthermore, mapping of the region around the *SNRPD3* variation contained a high number of mismatches and unknown bases, and the fact that *SNRPD3* is a splicesomal gene diminishes the reliability of this variation. The functional annotation of the rest of the genes containing a stopgain variation revealed several reproduction-related genes. *DAZAP1* is expressed most abundantly in the testis, but it does appear necessary for normal growth and development in mice (Hsu *et al.* 2008). Depletion of *DAZAP1* causes male and female sterility, underlining its importance in reproduction. Recent studies in mice have also shown the importance of *PPRC1* for early embryonic development (He *et al.* 2012). Homozygous deficient *PPRC1* mice fail to form egg cylinders and die before embryonic day 6.5 (He *et al.* 2012). *CETN2* is a calcium-binding protein and a structural component of the centrosome. In human cells, Centrin 2 depletion results in reduction in ciliogenesis (Graser *et al.* 2007), which implicates an effect on sperm tail formation and female reproduction through oviduct cilia. Other stopgain variations were found in *UNC45*, which has a role in HSP90-mediated myosin motor domain folding (Liu, Srikakulam and Winkelmann 2008), and therefore may have an impact on meat quality in pigs. P2RX4 has a role in the response of endothelial cells to changes in blood flow (Yamamoto *et al.* 2006), and ERCC8 is required for DNA repair (Henning *et al.* 1995). Mutations within *ERCC8* have been shown to cause Cockayne syndrome (Bertola *et al.* 2006; Cao *et al.* 2004; Henning *et al.* 1995; Ridley *et al.* 2005), which is characterized by growth failure, impaired development of the nervous system, photosensitivity, and premature aging (Knoch *et al.* 2012). However, understanding the influence of these polymorphisms on phenotype requires further investigation. None of the stopgain variations had the homozygous genotype for the mutated allele, but the stoploss variations allowed homozygosity (Table S6). Thus, the phenotypic effect of these variations is clearly less dramatic.

**Table 2:** Polymorphisms identified in the Finnish Large White

Coding	9510
Coding, splicing	7
Downstream	1333
Downstream, upstream	25
Intergenic	8667
Intronic	4343
Splicing	1187
UTR3	3855
UTR3, UTR5	4
UTR5	539
Upstream	503
Total	29,973

**Table 3:** Identified stopgain and stoploss variations within the Finnish Large White

Chr	Position	Identifier	Ref/Alt	DP	QD	Quality	Classification	Gene	Exon	HGVS Coding	HGVS Protein
2	77952555	?	G/A	13	19	165	Stopgain	*DAZAP1*	5	c.304C > T	p.Gln102*
4	107810302	?	C/A	46	26	1723	Stopgain	*MCL1*	2	c.92C > A	p.Ser31*
7	58576014	?	G/T	38	6	248	Stopgain	*UNC45A*	18	c.2320G > T	p.Glu774*
13	1095554	?	G/A	13	8	102	Stopgain	*ASTE1*	4	c.146G > A	p.Trp49*
14	33232916	?	T/A	19	8	166	Stopgain	*P2RX4*	13	c.1084A > T	p.Lys362*
14	53029449	?	A/T	129	5	784	Stopgain	*SNRPD3*	2	c.22A > T	p.Lys8*
14	122996299	?	C/A	16	6	96	Stopgain	*PPRC1*	5	c.1442C > A	p.Ser481*
16	42659625	?	C/T	13	8	105	Stopgain	*ERCC8*	4	c.321G > A	p.Trp107*
7	123586577	?	T/A	11	21	254	Stoploss	*DICER1*	22	c.4697A > T	p.*1566Leuext*?
8	124953125	?	A/T	14	28	391	Stoploss	*PPA2*	5	c.332A > T	p.*111Leuext*22
14	12523156	rs81212863	A/G	155	32	5198	Stoploss	*CLU*	3	c.124T > C	p.*42Argext*13
14	41268275	?	A/C	40	29	1542	Stoploss	*OAS1*	6	c.1076A > C	p.*359Serext*8
14	88489098	rs196953202	T/C	49	20	671	Stoploss	*ANXA11*	13	c.1324T > C	p.*442Argext*10
16	65096704	?	A/C	12	28	335	Stoploss	*MAT2B*	7	c.976T > G	p.*326Glyext*11
X	141373463	?	T/G	94	18	2304	Stoploss	*CETN2*	6	c.477A > C	p.*159Cysext*22

DP, read depth; QD, quality by depth.

When compared to the DE gene lists, 57 nonsynonymous SNPs were present in upregulated genes in the testis and 67 were present in the oviduct (Table S7). In the oviduct, the 67 genes were involved in 16 different biological processes, including, *e.g.*, reproduction, cellular component morphogenesis, and cell cycle. The polymorphisms in reproduction-related genes were confirmed by visualization with the Integrative genomics viewer (IGV) (Robinson *et al.* 2011; Thorvaldsdottir, Robinson and Mesirov 2013). Three oviduct genes (*FRAS1*, *TCF4*, and *PGR*) were associated with reproduction, and QTL regions for sow reproductive traits have been previously identified within these regions (Table S8). FRAS1 has been localized in embryonic epithelial basement membranes in the mouse (Chiotaki *et al.* 2007), and mutations within the gene have been shown to cause Fraser syndrome in humans (Hoefele *et al.* 2013; Ogur *et al.* 2011; Vogel *et al.* 2012). PGR, a progesterone receptor, is highly expressed in the ovary and in the oviduct, which makes it a good candidate gene for sow reproduction. Progesterone is critical for successful ovulation and for the multi-faceted functioning of the oviduct in mammalian reproduction (Akison and Robker 2012). Several nonsynonymous SNPs were identified within PGR (Table S8). TCF4 is a transcription factor and is widely expressed. In the testis, five upregulated genes (*ADAT1*, *SPAG6*, *PIWIL2*, *PKDREJ*, and *DNAH8*) with nonsynonymous variations had a classified role in reproduction. A QTL for male reproduction has only been identified for the genomic region around *DNAH8* (chromosome 7: 39,286,129-39,569,290). This region was associated with epididymis weight. In addition, a high number of additional QTL have been associated with this region (n = 232; http://www.animalgenome.org/). DNAH8 has a role in sperm motility and has been shown to be crucial for male fertility (Fossella *et al.* 2000; Olds-Clarke and Johnson 1993; Samant *et al.* 2002). The identified QTL for epididymis weight within the DNAH8 region may be influenced by a lower epididymal sperm count due to malformed sperm. Furthermore, *PKDREJ* appeared to contain several nonsynonymous SNPs, including two deleterious based on SIFT analysis (Table S8). *PKDREJ* is a male germ cell–specific polycystin, which is required for acrosome reaction during sperm–egg fusion (Butscheid *et al.* 2006; Sutton *et al.* 2006). *PKDREJ* has also been identified on the surface of ejaculated boar spermatozoa (Zigo *et al.* 2013). Thus, these variants may be considered strong candidate polymorphisms for boar fertility, although identification of a phenotypic effect requires further study.

### Validation of the reproduction-related SNPs and expression differences between the testis and oviduct

**Figure 5:**
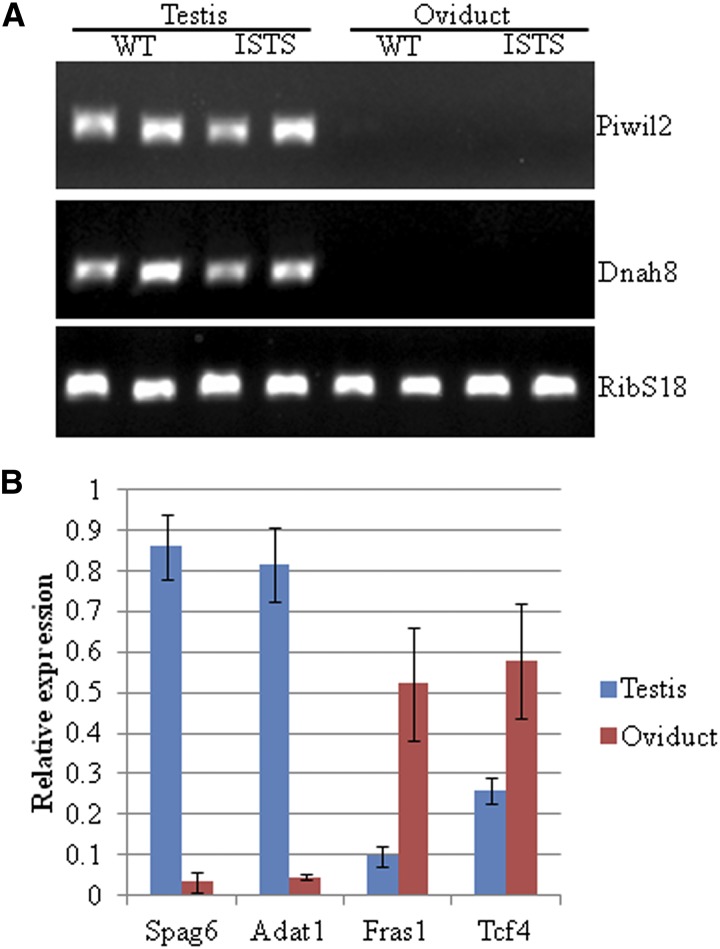
Differential gene expression of identified reproduction-related genes with polymorphisms in the testis and oviduct. (A) *PIWIL2* and *DNAH8* are predominantly expressed in the testis. (B) qPCR results of genes *SPAG6*, *ADAT1*, *FRAS1*, and *TCF4* confirm identified expression differences between the testis and oviduct in the RNAseq data.

Differential expressions identified in the RNAseq data were validated by RT-PCR of selected genes in the testis and oviduct tissue samples. For the validation, we selected DE genes associated with reproduction that contained nonsynonymous SNPs, because these genes represent potential candidate genes for an effect in reproduction traits. We analyzed the expression differences between the testis and oviduct samples for four genes (*FRAS1*, *TCF4*, *ADAT1*, and *SPAG6*) by qPCR and for two additional genes (*PIWIL2* and *DNAH8*) by RT-PCR and an agarose gel. All genes showed a similar expression pattern in the RT-PCR analysis, as detected by RNAseq (Figure 5). Testis-specific genes *ADAT1*, *SPAG6*, *PIWIL2*, and *DNAH8* exhibited none or extremely low expression in the oviduct. The genes with higher expression in the oviduct compared to the testis in the RNAseq data appeared to be present in the testis samples, but at a much lower level (Figure 5B). Furthermore, the polymorphisms within genes *FRAS1*, *ADAT1*, *SPAG6*, *DNAH8*, and *PGR* were confirmed by Sanger sequencing. Thus, these polymorphisms represent potential candidates for gene-assisted selection.

A possible effect of the validated SNPs was analyzed using the SIFT and CFSSP prediction tools. All nonsynonymous SNPs were tolerated, but the SIFT score indicated a possible effect on protein function due to the level of conservation of the amino acid sequence at the SNP location for *FRAS1*, *ADAT1*, *SPAG6*, and *PIWIL2* (SIFT score <0.35) (Table S8). The effect on protein secondary structure was also explored by CFSSP, which indicated a shift in the helix structure at the SNP position for *SPAG6* and *FRAS1* and the removal of a helix in *PIWIL2*. Although the effect of the identified SNPs on protein function and phenotypic differences is yet to be investigated, our data suggest possible causative mutations for differences in reproductive performance in the Large White pig population.

## Conclusions

The RNAseq technology applied in the present study provides new information regarding the extent of variation within reproduction-related genes in the testis and oviduct. In addition to previously annotated genes, we detected 55 previously unannotated gene orthologs in the pig based on blast analysis against the human, cow, and sheep genomes. The identified nonsynonymous mutations within the highly expressed genes in the testis and oviduct underline the possible identity of genes affecting the fertility in the Finnish Large White. Several stopgain variations were also detected highlighting the potential high-impact gene polymorphisms for reproductive and other genetic disorders. Furthermore, we validated seven nonsynonymous mutations in testis-specific or oviduct-specific genes, which underline the potential of these variants as candidates for selection in reproduction traits.
